# Supplementary material for: Mitochondrial fatty acid oxidation is stimulated by red light irradiation
Source: FEBS Lett. 2025 Oct 18;600(1):20–38. doi: 10.1002/1873-3468.70195 (PMC12793707; doi:10.1002/1873-3468.70195)
Supplement: Supplementary file 1 — Fig. S1. Spectral distributions of LEDs used for irradiation treatments. Fig. S2. Mitochondrial respiration is differentially modulated by different light wavelengths. Fig. S3. Moderate doses of red light (660 nm) increase mitochondrial respiration. Fig. S4. No changes in proteins related to fatty acid oxidation upon treated with Red light. Fig. S5. No effects on lipid content upon treatment with UVA, blue or green light. Fig. S6. No effects on lipid metabolic checkpoints upon treatment with UVA, blue or green light. Table S1. LED specifications and photons from each irradiator used. Table S2. LED specifications and photons of the Ethik irradiator used for red light treatments. [file FEB2-600-20-s001.pdf]

Supplementary material

Figure Legends

Figure S1. Spectral distributions of LEDs used for irradiation treatments: UVA (365 nm), blue light (450 nm), green light (517 nm), and red light (660 nm). Irradiance was measured as described in the methods section.

Figure S2. Mitochondrial respiration is differentially modulated by different light wavelengths. Cell oxygen consumption rates (OCR) and Extracellular Acidification Rates (ECAR) were quantified after 2 hours irradiation at 36 J/cm^2 with UVA (365 nm), blue light (450 nm), green light (517 nm), or red light (660 nm). Reserve (A), proton leak (B) and non-mitochondrial OCRs (C) as well as ECARs (D) were measured as described in Methods under basal conditions, followed by injection of oligomycin (oligo, 1 μM), CCCP (1 μM), and antimycin A plus rotenone (AA/Rot, 1 μM each). Results are means ± SD of three independent experiments; ns = > 0.1. One-way ANOVA followed by Dunnett.

Figure S3. Moderate doses of red light (660 nm) increase mitochondrial respiration. OCRs (A-C) and ECAR (D) were quantified under similar conditions to Fig. S2 after 2 hours irradiation at 6, 12, 36 and 150 J/cm^2 with red light. Results are expressed as mean ± SD of three independent experiments; ns = > 0.1, One-way ANOVA followed by Dunnett.

Figure S4. No changes in proteins related to fatty acid oxidation upon treated with Red light. Western blot analysis of protein levels was conducted in cells 2 hours post exposure to red light at different doses (0, 12, 36 J/cm^2). Extracts were collected and protein quantified by BCA. Carnitine palmitoyl transferases (CPT1/CPT2) (A, B), Electron-transfer-flavoprotein (ETFA) (C) and Pyruvate dehydrogenase (PDH) (D) where quantified. Results are expressed as means ± SD of four independent experiments; ns = > 0.1, One-way ANOVA followed by Dunnett.

Figure S5. No effects on lipid content upon treatment with UVA, blue or green light. Non-Esterified Free Fatty Acids (A), Triglycerides (B) and Cholesterol (C) where quantified in keratinocytes after 2 hours exposure to UVA (365 nm), blue light (450 nm), and green light (517 nm) at a single dose of 36 J/cm^2. Results are expressed as means ± SD of three independent experiments; ns = > 0.1, One-way ANOVA followed by Dunnett.

Figure S6. No effects on lipid metabolic checkpoints upon treatment with UVA, blue or green light. Western blot analysis of protein levels was conducted in cells 2 hours post exposure to UVA (365 nm), blue light (450 nm), and green light (517 nm) at a single dose of 36 J/cm^2. Extracts were collected and protein quantified by BCA. AMP-activated protein kinase (AMPK) (A), Acetyl-CoA carboxylase (ACC) (D), and their phosphorylated forms (B, E) were quantified, and the ratios of P-AMPK/AMPK (C) and P-ACC/ACC (F) were calculated. Results are expressed as means ± SD of four independent experiments; ns = > 0.1. One-way ANOVA followed by Dunnett.

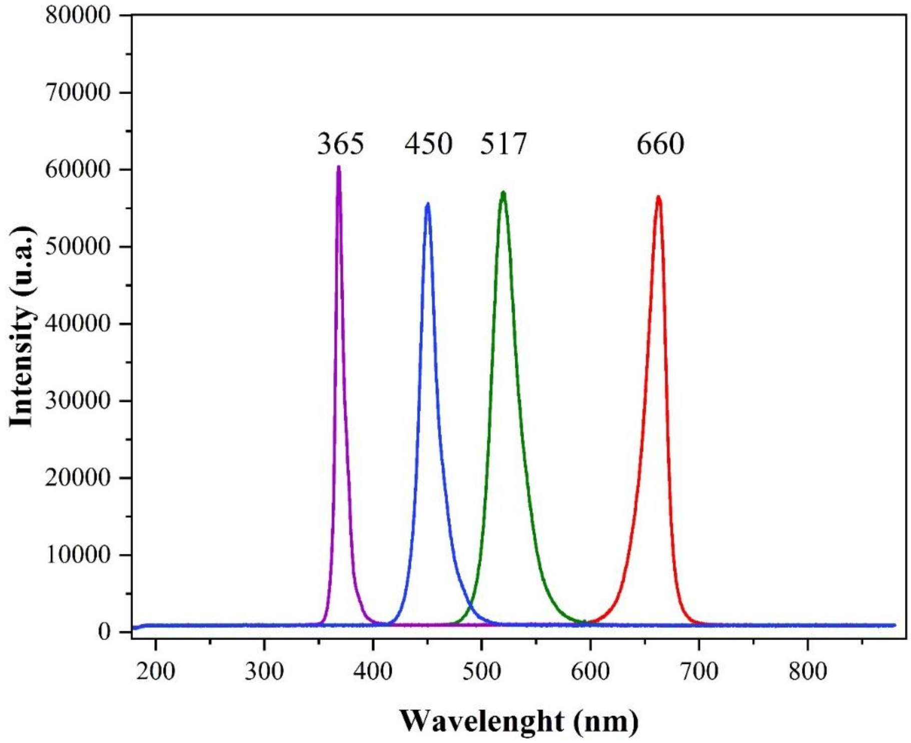

Herrera et al., Supplementary Figure S1

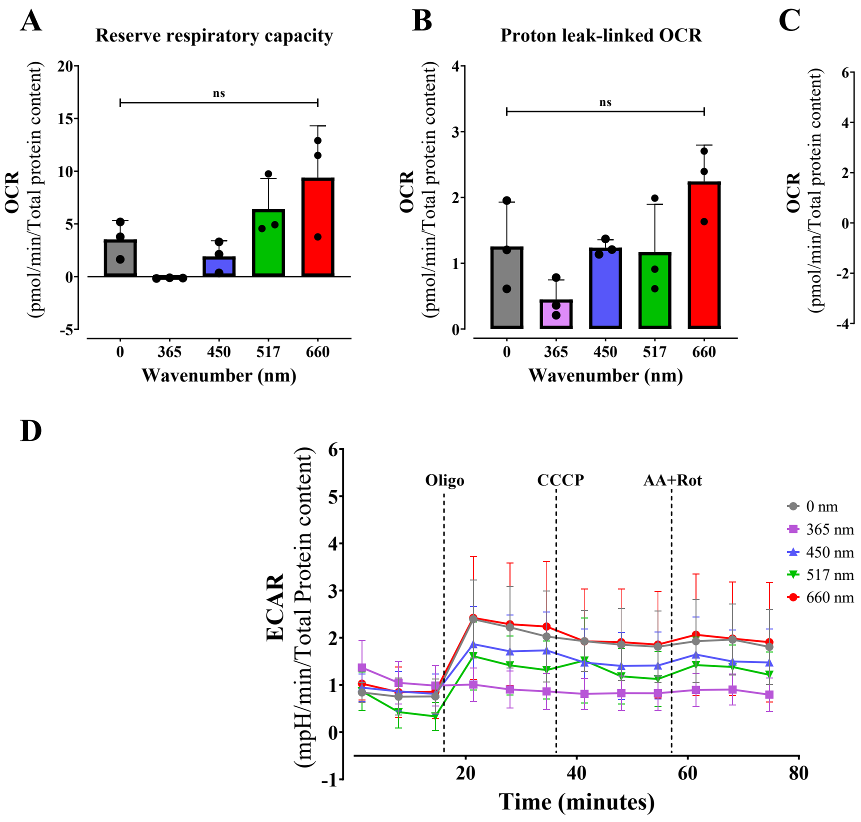

Herrera et al., Supplementary Figure S2

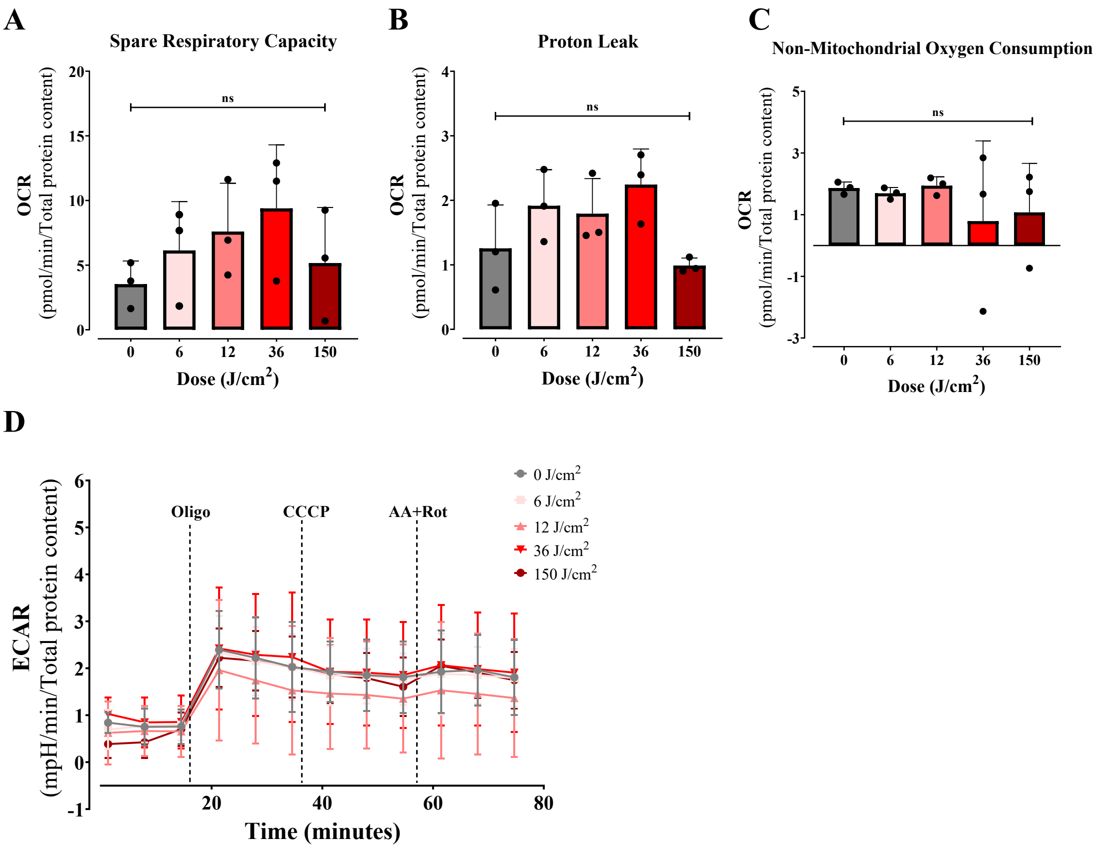

Herrera et al., Supplementary Figure S3

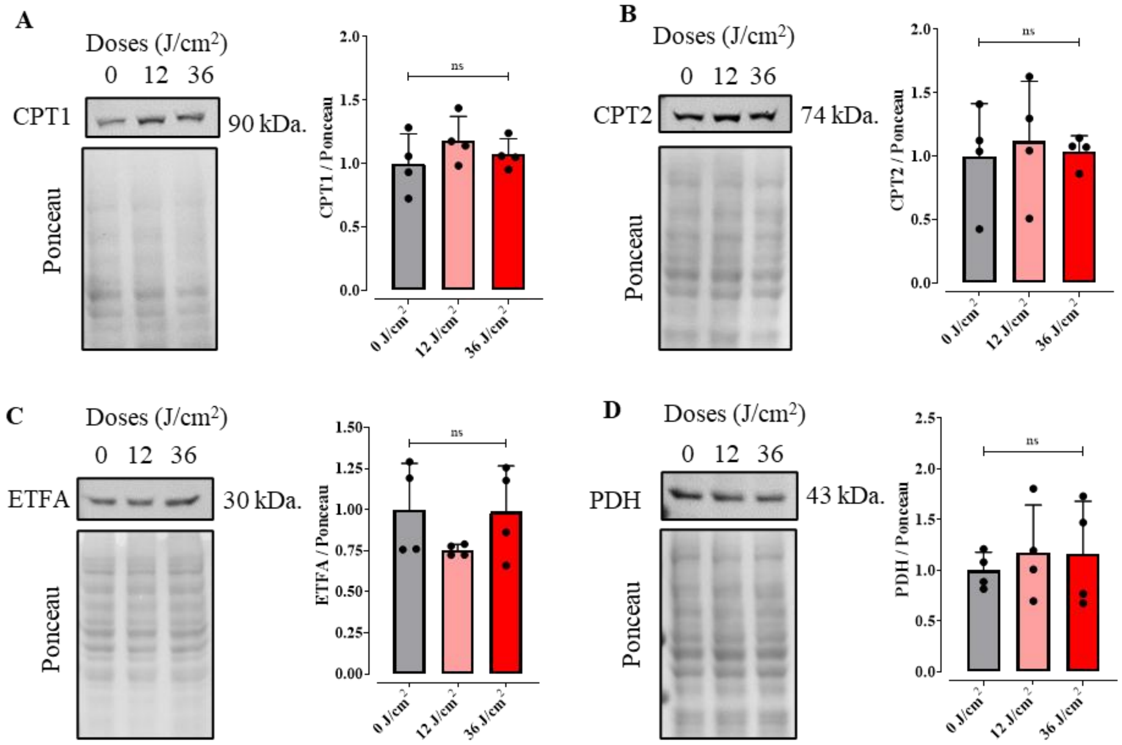

Herrera et al., Supplementary Figure S4

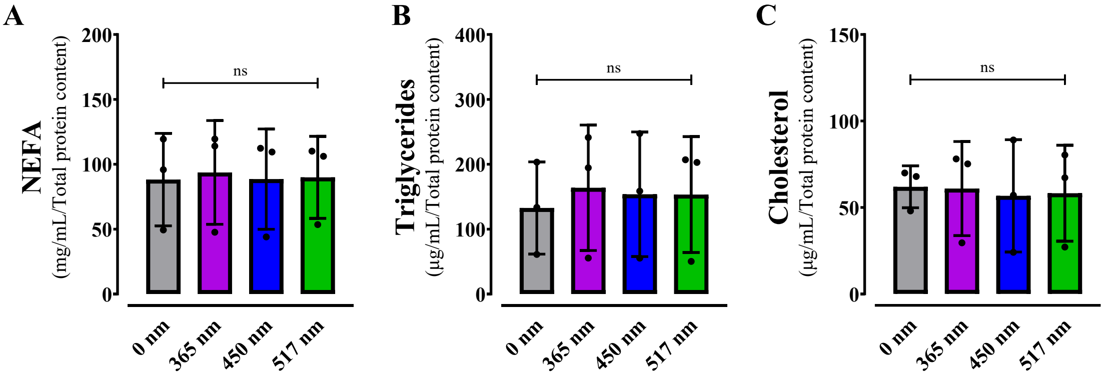

Herrera et al., Supplementary Figure S5

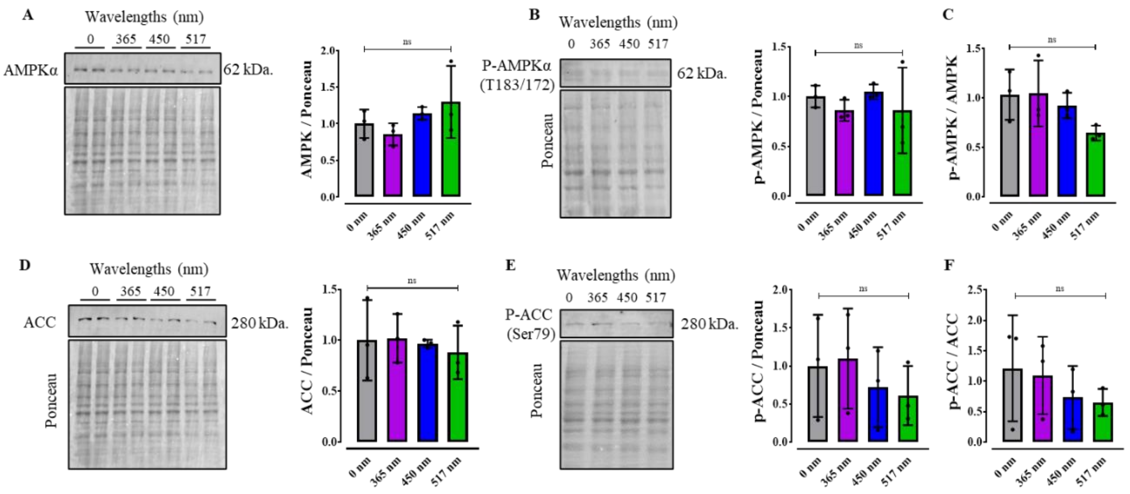

Herrera et al., Supplementary Figure S6

Supplementary tables

Table S1. LED specifications and photons from each irradiator used for UVA (365 nm), Blue light (450 nm), Green light (517 nm) and Red light (660 nm) with a dose of 36 J/cm^2. Energy was calculated using the formula E= hc/λ, where "h" is the Planck's constant, "c" is the speed of light in vacuum and λ is the wavelength.

Wavelength (m)	Intensity (mW)	Energy (J)	Number of photons / s	Dose (J/cm^2)	Area (cm^2)	Time (s)	Total number of photons
3,65 x 10^{-7}	2,16	5,45 x 10^{-19}	3,96 x 10^{-18}	36	0,283	4716,67	1,87 x 10^{-22}
4,50 x 10^{-7}	16,70	4,42 x 10^{-19}	3,78 x 10^{-19}	36	0,49	1056,29	3,99 x 10^{-22}
5,17 x 10^{-7}	13,00	3,85 x 10^{-19}	3,38 x 10^{-19}	36	0,49	1356,92	4,59 x 10^{-22}
6,60 x 10^{-7}	12,20	3,01 x 10^{-19}	4,05 x 10^{-19}	36	0,49	1445,90	5,85 x 10^{-22}

Table S2. LED specifications and photons of the Ethik irradiator used for red light treatments (660 nm) with the different doses (6, 12, 36 and 150 J/cm^2). Energy was calculated using the formula of E= hc/λ, where "h" is the Planck's constant, "c" is the speed of light in vacuum and λ is the wavelength.

Wavelength (m)	Intensity (mW)	Energy (J)	Number of photons / s	Dose (J/cm^2)	Area (cm^2)	Time (s)	Total number of photons
6,60 x 10^{-7}	12,20	3,01 x 10^{-19}	4,05 x 10^{-19}	6	0, 49	240,98	9,76 x 10^{-21}
6,60 x 10^{-7}	12,20	3,01 x 10^{-19}	4,05 x 10^{-19}	12	0,49	481,96	1,95 x 10^{-22}
6,60 x 10^{-7}	12,20	3,01 x 10^{-19}	4,05 x 10^{-19}	36	0,49	1445,90	5,85 x 10^{-22}
6,60 x 10^{-7}	12,20	3,01 x 10^{-19}	4,05 x 10^{-19}	150	0,49	6024,59	2,44 x 10^{-23}
